# Supplementary material for: Understanding Cervical Cancer Screening Attendance: Barriers and Facilitators in a Representative Population Survey
Source: Cancers (Basel). 2025 Feb 19;17(4):706. doi: 10.3390/cancers17040706 (PMC11853127; doi:10.3390/cancers17040706)
Supplement: Supplementary file 1 [file cancers-17-00706-s001.zip › cancers-3472370-supplementary.pdf]

P0. Age: ___________

Age framing
- 25-35 years
- 36-45 years
- 46-55 years
- 56-64 years

HEALTH-RELATED QUESTIONS

Q1. How would you describe your health?
- Very good
- Pretty good
- Neither good nor bad
- Pretty bad
- Very bad
- I don't know
- I'm not responding

Q2. Do you have a chronic illness (a disease that requires continuous treatment)?
- Yes
- No
- I'm not responding

Q2_1. Are you a smoker?
- Yes, I smoke regular cigarettes
- Yes, I smoke electronic cigarettes, products that heat tobacco
- No
- I'm not responding

Q3. Do you have a family doctor?
- Yes
- No
- I don't know
- I'm not responding

If YES
Q3_1. How frequently have you been to your family doctor over the past three years for problems with your personal health?
- Once every three months or more often
- Twice a year
- Once a year
- Less often than once a year
- I'm not responding

Q4_3. Do you currently use contraception and/or protection?
- Yes
- No
- I'm not responding

If YES
Q4_3_1. What kind of contraceptive and/or protective methods do you use (multiple choice)?
- Condom
- Contraceptive pill
- Intrauterine device (IUD)
- Calendar method (safe days)
- Withdrawal (Coitus interruptus)

- Sterilization
- Injections
- Others. Which?
- I'm not responding

If YES, the contraceptive pill
Q 4_3_1_COC. How long have you been using birth control pills?
- Less than five years
- More than five years
- I don't know
- I'm not responding

Q4_4_0. Have you been pregnant before?
- Yes
- No
- I'm not responding

Q4_4_1. If so, how many births have you had? ___________

Q4_4_1i. Birth classification
- No births
- One birth
- 2-5 births
- More than five births
- I'm not responding

Q 4_4_1prim. At what age did you have your first birth?
- Under 17
- 17-25 years
- 26-30 years
- Over 30 years
- I'm not responding

If you have been pregnant
Q4_4_4. At the moment, how many children do you have? ___________

Q4_4_2_1. Frame number of children
- No children
- One child
- 2-4 Children
- 5-7 Children
- Eight children or more

CERVICAL CANCER

Q5. Have you heard of cervical cancer?

- Yes
- No
- I'm not responding

If YES

Q5_0. As far as you know, what is cervical cancer?

- ___________
- I don't know
- I'm not responding

Q5_1. From what you know, what are the risk factors for cervical cancer? Risk factors increase a person's chances of having this condition.

- HPV infection
- More sexual partners
- Young age at first sexual intercourse
- Smoking
- Poor hygiene during menstruation
- Many tasks
- Using the contraceptive pill
- Increased number of sexually transmitted diseases
- Many abortions
- Genetic inheritance
- Many births
- Difficult births
- Cesarean section
- Decreased immunity of the body
- Poor nutrition
- Others. Which?
- I don't know
- I'm not responding

Q5_2. As far as you know, what are the symptoms (signs of) cervical cancer? (multiple choice)

- Bleeding between menstrual cycles
- Vaginal discharge with an unpleasant smell
- Vaginal or pelvic pain
- Bleeding after menopause
- Periods with more or longer bleeding than normal
- Pain during sexual intercourse
- No symptoms
- Others. Which?
- I don't know
- I'm not responding

Q5_3. As far as you know:

- Can cervical cancer be cured?
    - Yes
    - No
    - I'm not responding
- Can cervical cancer be prevented?
    - Yes
    - No
    - I'm not responding
- Is there treatment for cervical cancer?
    - Yes

- No
- I'm not responding

If it can be prevented
Q5_3_1. By what methods can it be prevented?
- Sexual abstinence (never having sex with anyone)
- Having sex with only one partner
- Use condoms
- Be vaccinated
- Have rigorous hygiene in the intimate area
- Participate in testing/screening from time to time
- Others. Which?
- I don't know
- I'm not responding

If it can be treated
Q5_3_2. By what methods can it be treated? (multiple choice)
- Ovum-type drugs inserted into the vagina
- Chemotherapy
- Radiotherapy
- Cauterization (destruction of affected tissue)
- Surgical (Hysterectomy, that is, complete removal of the uterus))
- Others. Which?
- I don't know
- I'm not responding

SCREENING FOR CERVICAL CANCER

Q6. Have you heard of a cervical cancer test?

- Yes
- No
- I'm not responding

If YES

Q6_1. Do you know what it is called?

- How? (Papanicolaou / Human Papilloma Virus-HPV Test) ____
- No
- I'm not responding

Q6_2. Where did you hear about the cervical cancer test? (multiple choice)

- Gynecologist
- Family doctor
- Nurse
- Community Nurse
- Sanitary Mediator
- Assistant Social
- Pharmacist
- Other categories of medical personnel, which include doctors of different specializations, etc.
- Friends
- Family/Rude
- TV
- Radio
- School/College/Work
- Leaflets/posters
- I don't know
- I'm not responding

Q7. At the family doctor's office, the doctor and/or nurse:

- Have they talked to you about the possibility of testing/screening for cervical cancer?
  - Yes
  - No
  - I'm not responding
- Did they give you a referral to your gynecologist for testing/screening?
  - Yes
  - No
  - I'm not responding
- Did they offer you a cervical cancer test at the office?
  - Yes
  - No
  - I'm not responding
- Did they provide you with information leaflets on testing/screening?
  - Yes
  - No
  - I'm not responding

Q8. Have you heard of the Papanicolaou test?

- Yes
- No
- I'm not responding

If YES

Q8_1. Where did you hear about the Papanicolaou test? (multiple choice)

- Gynecologist

- Family doctor
- Nurse
- Community Nurse
- Sanitary Mediator
- Assistant Social
- Pharmacist
- Other categories of medical personnel, which include doctors of different specializations, etc.
- Friends
- Family/Rude
- TV
- Radio
- School/College/Work
- Leaflets/posters
- I don't know
- I'm not responding

Q8_2. Have you ever taken this test?

- No
- Only once
- Two times
- Three times
- Four times
- Five times
- More than five times

If she took his test
Q8_2_1. When was the last time you took this test?

- This year
- Last year
- Two years ago
- Three years ago
- Five years ago
- More than five years ago
- I don't remember
- I'm not responding

Q8_2_2. Who took this test for you?

- Your family doctor (general practitioner)
- Another general practitioner in your community
- Your gynecologist
- A local gynecologist for whom you have received a referral from your family doctor
- A gynecologist from another locality for whom you have received a referral from your family doctor
- The mobile unit when it came to your community
- Somebody else. Who?
- I don't know
- I'm not responding

Q8_2_3. How often do you think you need to have a Pap smear?

- Once every six months
- Once a year
- Every three years
- Every five years
- Less often than five years
- I don't know
- I'm not responding

If she didn't take his test
Q8_2_4. Why have you never taken this test?
- I had no money
- I didn't have time
- I didn't know it was necessary
- I had to go too far
- No medical professional recommended this test to me
- I had no symptoms
- For religious reasons
- My family disapproves of such tests
- I was ashamed
- I was afraid of the result (to find out I had cancer)
- There's no point; if you have cancer, there's nothing you can do anyway
- Another answer. Which?
- I don't want to talk about it
- I'm not responding

Q9. Have you ever heard of the human papillomavirus, HPV?
- Yes
- No
- I'm not responding

If YES
Q9_1. As far as you know, how is this virus transmitted? (multiple choice)
- Through sexual intercourse
- Genetic/hereditary inheritance
- Through blood/medical instruments
- From mother to fetus
- Otherwise. How exactly?

Q9_2. As far as you know, what condition causes HPV?
- ___________
- I don't know
- I'm not responding

Q9_3. Where have you heard of HPV? (multiple choice)
- Gynecologist
- Family doctor
- Nurse
- Community Nurse
- Sanitary Mediator
- Assistant Social
- Pharmacist
- Other categories of medical personnel, which include doctors of different specializations, etc.
- Friends
- Family/Rude
- TV
- Radio
- School/College/Work
- Leaflets/posters
- I don't know
- I'm not responding

Q9_4. Have you ever had an HPV test (genotyping)?
- No
- Only once

- Two times
- Three times
- Four times
- Five times
- More than five times

Q9_5. Have you heard of an HPV vaccine?
- Yes
- No
- I'm not responding

If YES
Q9_5_1. Where did you hear about the HPV vaccine? (multiple choice)
- Gynecologist
- Family doctor
- Nurse
- Community Nurse
- Sanitary Mediator
- Assistant Social
- Pharmacist
- Other categories of medical personnel, which include doctors of different specializations, etc.
- Friends
- Family/Rude
- TV
- Radio
- School/College/Work
- Leaflets/posters
- I don't know
- I'm not responding

Q9_5_2. If you have a daughter between the ages of 11 and 14, would you vaccinate her against HPV?
- Yes
- No
- I'm not responding

If YES
Q9_5_3_1. Why so?
- ___________
- I don't know
- I'm not responding

If NOT
Q9_5_2_2. Why not?
- ___________
- I don't know
- I'm not responding

ATTITUDES

Q10. Please let me know whether or not you agree with the following statements

- I trust the medical services in my locality
  - Yes
  - No
  - I'm not responding
- Maintaining rigorous intimate hygiene can prevent cervical cancer
  - Yes
  - No
  - I'm not responding
- You can cure yourself of cervical cancer
  - Yes
  - No
  - I'm not responding
- Bleeding between periods is normal
  - Yes
  - No
  - I'm not responding
- Taking a Papanicolaou test is expensive
  - Yes
  - No
  - I'm not responding
- I will not get cervical cancer
  - Yes
  - No
  - I'm not responding
- If someone has cervical cancer, I keep my distance from the woman
  - Yes
  - No
  - I'm not responding
- Taking an HPV test is expensive
  - Yes
  - No
  - I'm not responding
- A regular gynecological check-up is necessary
  - Yes
  - No
  - I'm not responding
- After starting sexual life, the HPV vaccine (against human papillomavirus) is no longer given
  - Yes
  - No
  - I'm not responding
- If I got cervical cancer, I would have access to treatment
  - Yes
  - No
  - I'm not responding

NATIONAL TESTING (SCREENING) PROGRAM FOR CERVICAL CANCER

Q11. Have you heard of the national cervical cancer testing/screening program?

- Yes
- No
- I'm not responding

If YES
Q11_1. Where did you hear about this program? (multiple choice)

- Gynecologist
- Family doctor
- Nurse
- Community Nurse
- Sanitary Mediator
- Assistant Social
- Pharmacist
- Other categories of medical personnel, which include doctors of different specializations, etc.
- Friends
- Family/Rude
- TV
- Radio
- School/College/Work
- Internet
- Social media - Facebook, Twitter, etc.
- Leaflets/posters
- I don't know
- I'm not responding

Q12. The national cervical cancer testing/screening program addresses all women in Romania aged between 25 and 64, regardless of whether they are insured or not. It involves taking a test (Papanicolaou or HPV) by a health specialist and receiving the results, and the services are free. Would you like to participate in this program?

- I have already participated
- Yes, I would like to participate
- No
- I'm not responding

Q12a. For the program that involves the activity of collecting a test (Papanicolaou or HPV) to detect a possible cervical cancer early, what term do you find easy to understand for everyone?

- National Screening Program for Cervical Cancer
- National Testing Program for Cervical Cancer
- Doesn’t matter
- I don't know
- I'm not responding

Q13. How should you be invited to participate in this program?

- Through your family doctor
- Through community health care
- By letter at home
- By SMS/internet
- Through pharmacists
- Through flyer in mailbox
- Another method. Which?
- I don't know
- I'm not responding

Q14. What are the factors that would convince you to participate in this program? (multiple choice)
- Collection of samples should be done near your home
- Be invited to the program along with people of the same age from your community
- Explain each step before testing
- Know more about cervical cancer
- Know more about how these tests are collected
- Another. Which?
- I don't know
- I'm not responding

If YES to Q12
Q12_1. Who would you prefer to collect your test (Papanicolaou or HPV)?
- Family doctor
- Nurse specialized in gynecology
- Gynecologist
- It doesn’t matter who
- I'm not responding

Q12_2. Who would you prefer to have your test (Papanicolaou or HPV)?
- A woman
- A man
- I'm not responding

Q12_3. If you had the full guidance on how to collect your HPV test, would you agree to the option of harvesting it yourself at home?
- Yes
- No
- I'm not responding

Q12_4 Where would you prefer the test to be taken?
- At the family doctor's office
- At the gynecology office
- The mobile unit coming to the community
- Would you instead take the test yourself, at home
- Indifferent
- I'm not responding

Q12_5. What are the reasons why you would not want to participate in this program of early testing and detection of cervical cancer?
- Negative opinions about the behavior of medical professionals
- Fear that you won't have enough privacy
- Shame
- Lack of time
- Difficulties in leaving the household
- Fear that I might have cancer
- There's no point if you have cancer; there's nothing you can do anyway
- My partner would disagree
- Others. Which?
- I don't know
- I'm not responding

Q14. Do you belong to any or more of the following groups? (multiple choice)
1. You have an income below the minimum / you are unemployed / don’t have a working place
2. You are not insured at the National Health Insurance House
3. Work in agriculture
4. You lived for a while in a foster home/child protection system

5. You are Roma
6. You have disabilities
7. You have children with disabilities
8. You are addicted to alcohol/other toxic substances
9. You are in the records of probation services
10. You are a victim of domestic violence
11. You have been a victim of human trafficking
12. Raising a child alone
13. None of the above
14. I'm not responding

We are at the end of the questionnaire; I only have a few more questions. Please respond to them only if you feel comfortable.

Q4. At what age did you have your first sexual intercourse?

- Option A: ≤14 nor
- Option B: 15-18
- Option C: 18-24
- Option D: 25-30 and
- Option A: ≥ 31
- Option F: Never
- I'm not responding

Q4_2. How many sexual partners have you had since you started having sex?

- Option A: 1-3 partners
- Option B: Between 4-7 partners
- Option C: More than 7 partners
- I don't know/I don't remember
- I'm not responding

SOCIO-DEMOGRAPHIC

P1. Ethnicity:
- Romanian
- Hungarian
- Rroma
- German
- Another ethnicity. Which? ___________
- I'm not responding

P2. Your religion:
- Christian: Roman Catholic, Greek Catholic
- Christian: Protestant, Anglican, Evangelical, Jehovah's Witnesses, Pentecostal, etc.
- Christian: Orthodox
- Islamic
- Mosaic/Jewish
- Secular/Non-Religious/Agnostic/Atheist/No Religion
- Other religion. Which? ___________
- I'm not responding

P3. The last school graduated:
- No school
- Primary school (grades 1 - 4)
- Middle school (grades 5 - 8)
- Apprenticeship school/ first level of high school (grades 9 - 10)
- Vocational school
- Highschool (grades 9 – 12)
- School foremen
- Post-secondary school
- Short-term university/college
- Long-term university
- Postgraduate studies (master, doctorate)
- I'm not responding

P4. Your occupation:
- Self-employed
- I am a business owner/entrepreneur
- Full-time employee
- Part-time employee
- Student
- Unemployed housewife
- Retired
- Childcare leave/Pre/post-natal leave
- Unemployed, temporarily unemployed
- Other situation. Which? ___________
- I'm not responding

P5. Your personal income, net, last month, regardless of its source:
- No income
- Less than 1000 lei
- 1001-2000 lei
- 2001-3000 lei
- 3001-4000 lei
- More than 4001 lei
- I'm not responding

P6. Residence environment:
- Urban
- Rural
- I'm not responding

P7. The county where you live:

____

P8. Development region:
- North-East
- South-East
- South-Muntenia
- South-West Oltenia
- West
- North-West
- Center
- Bucharest - Ilfov
- I'm not responding
